# Toxic Injury to Muscle Tissue of Rats Following Acute Oximes Exposure

**DOI:** 10.1038/s41598-018-37837-4

**Published:** 2019-02-06

**Authors:** Vesna Jaćević, Eugenie Nepovimova, Kamil Kuča

**Affiliations:** 1 National Poison Control Centre, Military Medical Academy, Belgrade, Serbia; 2 Faculty of Medicine of the Military Medical Academy, University of Defence, Belgrade, Serbia; 3 Department of Chemistry, Faculty of Science, University of Hradec Kralove, Hradec Kralove, Czech Republic

## Abstract

Therapeutic application of newly developed oximes is limited due to their adverse effects on different tissues. Within this article, it has been investigated which morphological changes could be observed in Wistar rats after the treatment with increasing doses of selected acetyl cholinesterase reactivators - asoxime, obidoxime, K027, K048, and K075. Subsequently, heart, diaphragm and musculus popliteus were obtained for pathohistological and semiquantitative analysis 24 hrs and 7 days after *im* administration of a single dose of 0.1 LD_50_, 0.5 LD_50_, and 1.0 LD_50_ of each oxime. Different muscle damage score was based on an estimation scale from 0 (no damage) to 5 (strong damage). In rats treated with 0.1 LD_50_ of each oxime, muscle fibres did not show any change. The intensive degeneration was found in all muscles after treatment with 0.5 LD_50_ of asoxime and obidoxime, respectively. Acute toxic muscle injury was developed within 7 days following treatment with 0.5 LD_50_ and 1.0 LD_50_ of each oxime, with the highest values in K048 and K075 group (P < 0.001 vs. control and asoxime), respectively. The early muscle alterations observed in our study seem to contribute to the pathogenesis of the oxime-induced toxic muscle injury, which probably manifests as necrosis and/or inflammation.

## Introduction

Acetylcholinesterase (AChE) reactivators, called oximes, were developed for the treatment of organophosphorus poisoning (OP)^1–4^. Exposure to organophosphorus compounds (OPC) has become common due to their use in agriculture as pesticides and the increased threat of nerve agent (NA) misuse during military conflicts and by terrorists^5,6^.

OPC inhibit an enzyme called acetylcholinesterase (AChE, E.C. 3.1.1.7), catalyses the breakdown of a neurotransmitter acetylcholine at the synaptic clefts. After its inhibition, AChE is unable to guarantee its physiological role causing acetylcholine accumulation, cholinergic receptors overstimulation and at the end cholinergic crisis^7,8^.

Due to the fact, that toxic effect of NA manifests very quickly (few minutes after intoxication), also approaches how to counteract these intoxications should be very fast by using antidotal treatment^9,10^. Antidotal treatment involves administration of anticholinergic drugs, AChE reactivators and anticonvulsants^11,12^.

**Figure 1:**
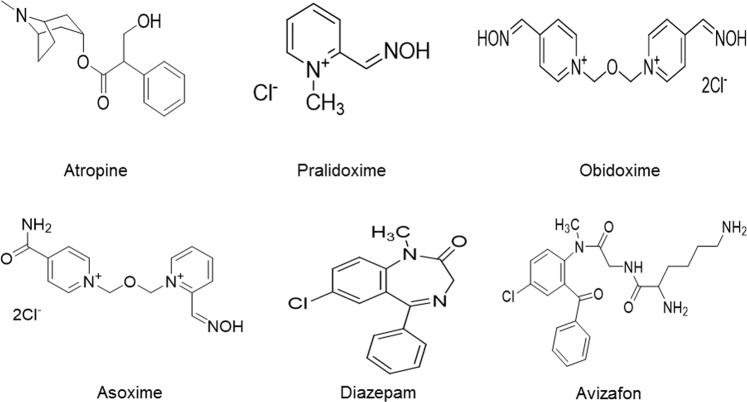
Chemical structure of antidotal treatment components.

Of these three components of antidotal therapy, AChE reactivators are researched very extensively because of their limited effects (Fig. 1).

The first and the major limitation has no universality. It means, that there is no reactivator able to treat sufficiently intoxications caused by different NA^4,12–14^. Another very important drawback of reactivators currently available on the market its low penetration into the brain and overall low central activity^15,16^. Moreover, asoxime (oxime HI-6), the best reactivator available on the market, is insufficiently stable in water, therefore must be used dry-wet auto-injectors as the appropriate application form must be used^17,18^. Due to this fact, many research groups throughout the world were designed and evaluated modern drug candidates acting as AChE reactivators, which could possibly replace the standard ones^19–25^.

Within this contribution, we turned our attention to an important task - the toxic effect of AChE reactivators themselves. Although they could help in case of poisoning and save the lives of victims, they are still xenobiotics, which could cause a harmful effect. Generally, if regulatory authorities, such as FDA, consider any new drug candidate to enter the market, the acute toxicity and adverse effect should be well researched prior to their approval^26^. In case of AChE reactivators, it is known, that they behave as weak inhibitors of AChE^27,28^. Furthermore, they interact with both types of cholinergic receptors^29–31^. Many cytotoxicities and *in vivo* toxicity studies on AChE reactivators have been published recently^32–34^, but there is a dearth of studies available concerning preclinical toxicology.

Regarding to these findings, acute toxicity screening and broad histopathological study focused on muscle morphological changes caused by AChE reactivators were performed to bring a new piece of the puzzle into the problematic of reactivators’ toxicity.

## Results

### Acute toxicity

(i). Asoxime. Asoxime was given in doses of 500, 600, 700 and 800 mg/kg *im*. Calculated LD_50_ value of asoxime is 673.85 mg/kg *im* (Table 1a). The first lethal effects of asoxime occurred after giving a dose of 600 mg/kg (2 out of 5), and 700 mg/kg (4 out of 5), respectively. After a single dose of 800 mg/kg of asoxime, 100% mortality rate was noticed.

**Table 1:** Effects of different oximes on 24 hrs survival in rats.

Oxime	Dose (mg/kg *im*)	Total number dead/treated	LD_50_ (mg/kg)	95% confidence limit
a) asoxime	500	0/5	626.38	583.96–671.89
	600	2/5		
	700	4/5		
	800	5/5		
b) obidoxime	100	0/5	163.97	142.57–188.57
	150	2/5		
	200	4/5		
	300	5/5		
c) K027	500	0/5	664.40	619.64–714.07
	600	1/5		
	700	3/5		
	800	5/5		
d) K048	100	0/5	229.39	164.15–320.56
	200	2/5		
	300	3/5		
	400	5/5		
e) K075	50	0/5	81.53	76.89–93.76
	75	2/5		
	100	3/5		
	150	5/5		

LD_50_ was calculated according to Litchfield & Wilcoxon.
(ii). Obidoxime. Obidoxime was given in increasing doses (100, 150, 200 and 300 mg/kg) by *im* route. Calculated LD_50_ value of obidoxime is 191.72 mg/kg *im* (Table 1b). The first lethal effects of obidoxime occurred after giving a dose of 150 mg/kg (2 out of 5) and 200 mg/kg (4 out of 5), respectively. A single dose of 300 mg/kg of obidoxime was induced 100% mortality rate.
(iii). K027. K027 was given in doses of 500, 600, 700 and 800 mg/kg *im*. Calculated LD_50_ value of K027 is 657.64 mg/kg *im* (Table 1c). The first lethal effects of K027 occurred after giving a dose of 600 mg/kg (1 out of 5), and 700 mg/kg (3 out of 5), respectively. After a single dose of 800 mg/kg of K027, 100% mortality rate was noticed.
(iv). K048. K048 was given in increasing doses (100, 200, 300 and 400 mg/kg) by *im* route. Calculated LD_50_ value of K048 is 234.69 mg/kg *im* (Table 1d). The first lethal effects of K048 occurred after giving a dose of 200 mg/kg (2 out of 5) and 300 mg/kg (3 out of 5), respectively. The single dose of 400 mg/kg of K048 was induced 100% mortality rate.
(v). K075. K075 was given in doses of 25, 50, 75 and 100 mg/kg *im*. Calculated LD_50_ value of K075 is 73.45 mg/kg *im* (Table 1e). The first lethal effects of K075 occurred after giving a dose of 50 mg/kg (2 out of 5), and 75 mg/kg (3 out of 5), respectively. The highest single dose of K075 (100 mg/kg *im*) was induced 100% mortality rate.

### The general condition of the experimental animals

The noticeable signs of acute toxicity did not show when animals exposed to 0.1LD_50_ or 0.5LD_50_ dose of different oximes. The clinical signs and symptoms were observed only in rats following 1.0 LD_50_ dose of each oxime exposure. The animals treated with 1.0 LD_50_ dose of each oxime showed muscle pain, tenderness, or weakness, and tremors during the completely experimental period.

### Pathohistological and semiquantitative analysis of the hearts of the experimental animals

In all groups treated with 0.1 LD_50_ of different oximes, single myocardial cells with intracellular oedema and normal nucleus were seen. A few, mild foci of perivascular cell infiltrates were observed in animals that received obidoxime or K075, only. In these groups, the CDS values were not significantly different from the other oxime-treated groups, as well as from the control ones, during the whole study period (Table 2a).

**Table 2:** The effects of different oximes (0.1 LD_50_, 0.5 LD_50_ or 1.0 LD_50_) on the degree of cardiac alterations - cardiac damage score (CDS) 24 hrs and 7 days after their administration.

Treatment	Days after treatment	CDS (5 hearts/group × 6 slices/heart) $\bar{{\boldsymbol{\times }}}$ ± S.D.		
		a) 0.1 LD_50_	b) 0.5 LD_50_	c) 1.0 LD_50_
control	1	0.13 ± 0.35	0.13 ± 0.35	0.13 ± 0.35
	7	0.17 ± 0.38	0.17 ± 0.38	0.17 ± 0.38
asoxime	1	0.13 ± 0.35	1.10 ± 0.71 **a**^**3**^	1.76 ± 0.68 **a**^**3**^
	7	0.20 ± 0.41	2.07 ± 0.69 **a**^**3**^**c**^**2**^	2.87 ± 0.78 **a**^**3**^**c**^**2**^
obidoxime	1	0.37 ± 0.49	1.93 ± 0.69 **a**^**3**^**b**^**3**^	2.70 ± 0.70 **a**^**3**^**b**^**3**^
	7	0.23 ± 0.45	3.00 ± 0.74 **a**^**3**^**b**^**3**^	3.77 ± 0.77 **a**^**3**^**b**^**3**^**c**^**3**^
K027	1	0.17 ± 0.38	1.67 ± 0.66 **a**^**3**^	2.53 ± 0.51 **a**^**3**^
	7	0.20 ± 0.41	2.80 ± 0.76 **a**^**3**^**c**^**3**^	3.33 ± 0.47 **a**^**3**^**b**^**3**^
K048	1	0.17 ± 0.38	2.03 ± 1.54 **a**^**3**^**b**^**3**^	3.50 ± 0.73 **a**^**3**^**b**^**3**^
	7	0.27 ± 0.45	3.73 ± 0.69 **a**^**3**^**b**^**3**^**c**^**3**^	4.40 ± 0.72 **a**^**3**^**b**^**3**^**c**^**3**^
K075	1	0.47 ± 0.51	2.16 ± 0.59 **a**^**3**^**b**^**3**^	3.43 ± 0.82 **a**^**3**^**b**^**3**^
	7	0.30 ± 0.47	3.47 ± 0.82 **a**^**3**^**b**^**3**^**c**^**3**^	4.07 ± 0.86 **a**^**3**^**b**^**3**^**c**^**2**^

Statistical evaluation was performed using Kruskall-Wallis test. **a**^**3**^ - P < 0.001 vs. control group; **b**^**3**^ - P < 0.001 vs. asoxime-treated group; **c**^**2**^, **c**^**3**^ - P < 0.01, 0.001 vs. 24 hrs.

**Figure 2:**
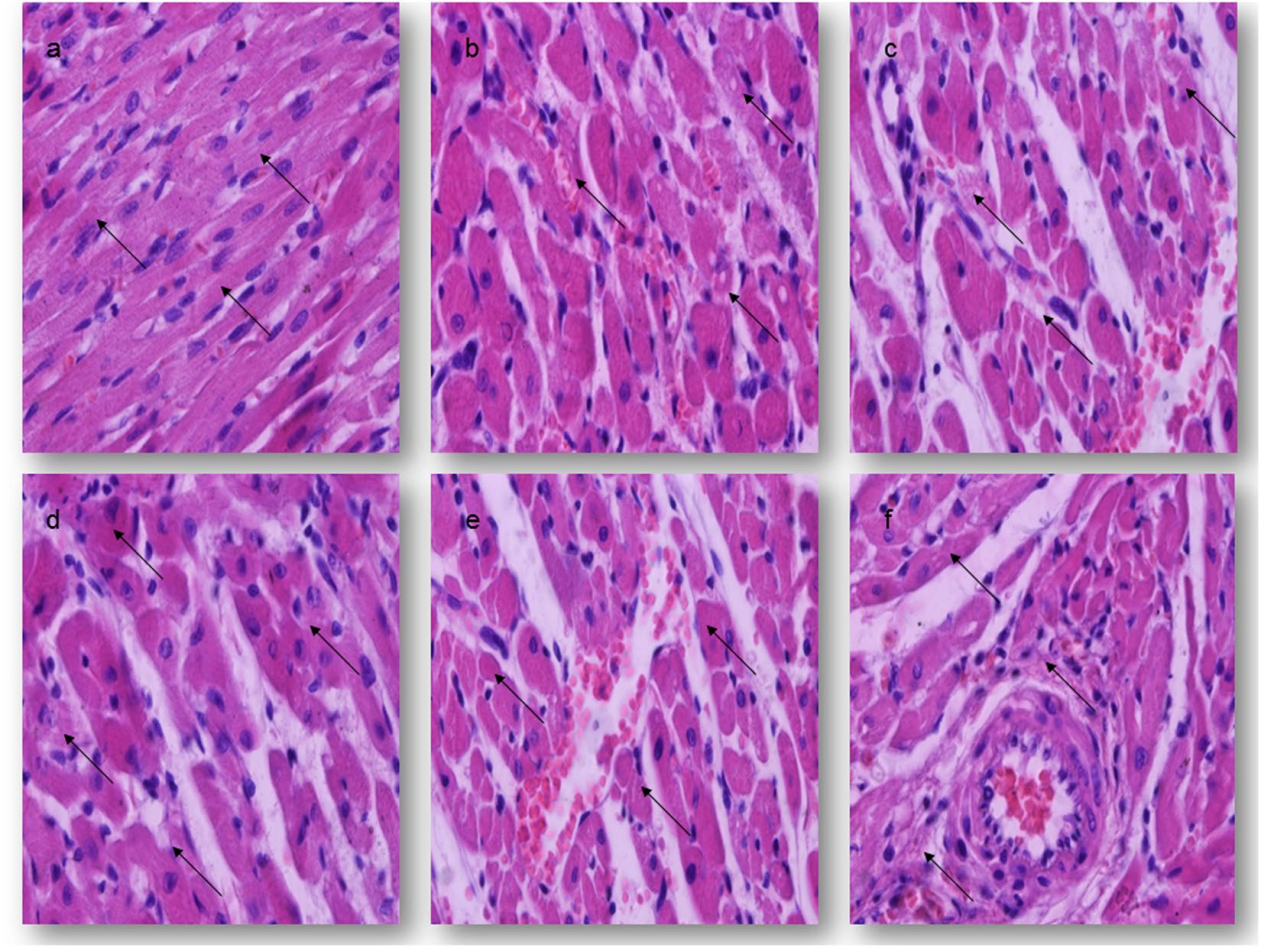
Light micrographs of the cardiac lesions of rats 7 days after treatments; H&E stain; magnification 400×; (**a**–**c**) −0.5 LD_50_ *im*; (**d**–**f**) −1.0 LD_50_ *im*. (**a**) The control group, normal histological structure of the cardiac myofiber; (**b**) The K048-treated group, moderate myofiber degeneration and focal haemorrhages; (**c**) The K075-treated group, focal fragmentation of myofibrils and accumulation of neutrophils and macrophages; (**d**) The asoxime-treated group, marked myofibril degeneration with prominent nucleoli; (**e**) The K048-treated group, severe myofibril degeneration and diffuse haemorrhage; (**f**) The K075-treated group, myofibril lysis, hyalinisation and cellular infiltration.

Myocardial alterations detected in all animals treated with 0.5 LD_50_ of different oximes were ranging from cytoplasm degeneration to focal necrosis of the small group of myocardial fibres and included moderate vascular changes, too. In the groups of animals treated with asoxime, sacrificed 24 hrs after application, degenerative changes were predominant, including intracellular oedema or discrete cytoplasmic vacuolisation with normal nuclei (Fig. 2a). Mild oedema and hyperaemia with focal cellular infiltration were presented in the epicardium and in the pericardium. The smallest CDS value of 1.10 ± 0.71 was established in asoxime-treated rats. This value was significantly different from those obtained in the control group. On the other hand, in the groups of animals treated with obidoxime, K048 or K075, the presence of moderate myofibril degeneration, hyperaemia, haemorrhages and tissue accumulation of neutrophils and macrophages were noticed focally. Fragmentation of muscle fibres was observed in about 20–30 per cent of the heart’s tissue. The degree of haemorrhages varied from a few erythrocytes to massive haemorrhagic foci in the interstitium that separates the bundles of the muscle fibres (Fig. 2b and c). The highest CDS value of 2.16 ± 0.59 was established in rats treated with K075, sacrificed after day 1 of the experiment. In addition, the CDS values of obidoxime, K048 and K075-treated rats were significantly higher from those obtained in the asoxime-treated rats and the control ones (P < 0.001). The frequency and severity of cardiac lesions significantly increased with time over a period of 1 to 7 days in all oxime-treated groups (P < 0.01 and P < 0.001, respectively) (Table 2b).

A single administration of a median lethal dose of asoxime, obidoxime, K027, K048 or K075 caused severe, diffuse and massive degenerative and vascular alterations in all treated animals. Affected muscle fibres had extensive sarcoplasm vacuolisation or pale eosinophilic sarcoplasm and lacked cross-striations. In the majority of these irregular, round to ovoid cells nuclear polymorphism was present, with large, round to rectangular shapes and prominent nucleoli. Retractile, slightly basophilic material was deposited at one pole of the nucleus. These affected areas were observed in the subepicardium, in the myocardium and in the endocardium in all treated rats, sacrificed after 24 hrs. Thickening of the blood vessels with vacuolisation of the endothelial cells was observed, too. The most striking findings were the presence of massive and diffuse haemorrhages, interstitial oedema, multifocal areas of myofibril lysis, and diffuse cellular infiltration consisting of neutrophils, monocytes and macrophages. The interstitial haemorrhages appeared uniformly in each of the sections examined, and were located in the middle myocardial or the subendocardial areas (Fig. 2d–f). The smallest CDS was evaluated in the asoxime-treated rats (1.76 ± 0.68). These values were significantly different from the control group (P < 0.001). Described pathohistological changes were the most intensive in the heart of rats sacrificed on day 7 of the study. The CDS values were significantly higher from those obtained on the first day of the study (P < 0.01 and P < 0.001, respectively). During the completely experimental period, the difference between the control and the oxime-treated groups was statistical significant (P < 0.001) (Table 2c).

### Pathohistological and semiquantitative analysis of the diaphragm of the experimental animals

**Figure 3:**
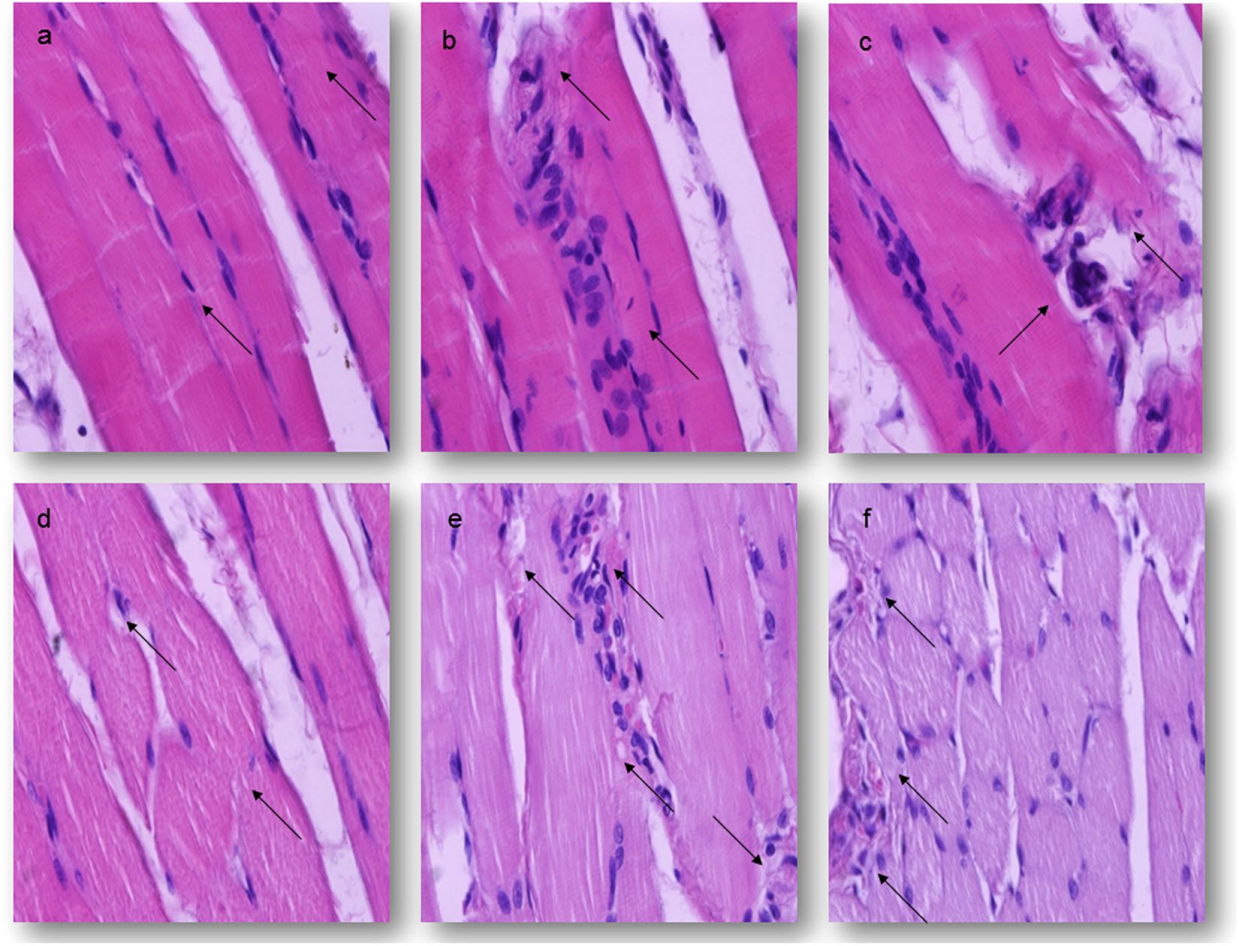
Light micrographs of the diaphragm lesions of rats 7 days after treatments. H&E stain. Magnification 400×. (**a**,**b**) −0.5 LD_50_ *im*; (**c**,**d**,**e**) −1.0 LD_50_ *im*; (**a**) The control group, normal histological structure of the diaphragm; (**b**) The K048-treated group, myofiber degeneration and cellular infiltration; (**c**) The K075-treated group, lack of cross-striations of muscle fiber and cellular infiltration; (**d**) The obidoxime-treated group, vacuolar degeneration of muscle fiber, moderate hyperaemia and oedema; (**e**) The K048-treated group, severe myopathy characterized by hyalinisation, vacuolization, atrophy and cellular infiltration (**f**) The K075-treated group, multifocal myofiber degeneration and necrosis characterized by discoid changes, myofibril lysis, hyalinisation, and cellular infiltration.

The histological features of the diaphragm fibres in the groups of animals treated with 0.1 LD_50_ of different oximes were similar to the control ones (Fig. 3a), during the completely experimental period. Oval nuclei were normal and located peripherally, just near the sarcolemma. Mild perivascular cell infiltrations were present on the external wall of dilatated blood vessels. In the same diaphragm tissue section, focal and discrete oedema and hyperaemia of the endomysium could be seen, too. These morphological changes were the most frequent in the group of rats treated with K075 (0.37 ± 0.61), but not significantly different from the other oxime-treated group or the control ones, during the whole study (Table 3a).

**Table 3:** The effects of different oximes (0.1 LD_50_, 0.5 LD_50_ or 1.0 LD_50_) on the degree of diaphragm alterations - diaphragm damage score (DDS) 24 hrs and 7 days after their administration.

Treatment	Days after treatment	DDS (5 diaphragm/group × 6 slices/diaphragm) $\bar{{\boldsymbol{\times }}}$ ± S.D.		
		a) 0.1 LD_50_	b) 0.5 LD_50_	c) 1.0 LD_50_
control	1	0.13 ± 0.35	0.13 ± 0.35	0.13 ± 0.35
	7	0.17 ± 0.38	0.17 ± 0.38	0.17 ± 0.38
asoxime	1	0.20 ± 0.41	1.53 ± 0.51 **a**^**3**^	1.80 ± 0.61 **a**^**3**^
	7	0.17 ± 0.38	2.33 ± 0.63 **a**^**3**^**c**^**3**^	2.80 ± 0.67 **a**^**3**^**c**^**3**^
obidoxime	1	0.23 ± 0.43	1.60 ± 0.50 **a**^**3**^	2.40 ± 0.62 **a**^**3**^
	7	0.20 ± 0.47	2.60 ± 0.55 **a**^**3**^**c**^**3**^	3.53 ± 0.94 **a**^**3**^**c**^**3**^
K027	1	0.10 ± 0.31	1.10 ± 0.66 **a**^**3**^	1.46 ± 0.51 **a**^**3**^
	7	0.20 ± 0.41	2.20 ± 0.71 **a**^**3**^**c**^**3**^	3.00 ± 0.73 **a**^**3**^**c**^**3**^
K048	1	0.13 ± 0.35	2.63 ± 0.61 **a**^**3**^**b**^**3**^	3.30 ± 0.70 **a**^**3**^**b**^**3**^
	7	0.23 ± 0.43	3.17 ± 0.71 **a**^**3**^	3.97 ± 0.76 **a**^**3**^**b**^**3**^
K075	1	0.37 ± 0.61	2.40 ± 0.62 **a**^**3**^**b**^**3**^	3.40 ± 0.62 **a**^**3**^**b**^**3**^
	7	0.27 ± 0.45	3.40 ± 0.76 **a**^**3**^**b**^**3**^**c**^**3**^	4.13 ± 0.50 **a**^**3**^**b**^**3**^**c**^**1**^

Statistical evaluation was performed using Kruskall-Wallis test. **a**^**3**^ - P < 0.001 vs. control group; **b**^**3**^ - P < 0.001 vs. asoxime-treated group; **c**^**1**^, **c**^**3**^ - P < 0.05, 0.001 vs. 24 hrs.

In animals treated with 0.5 LD_50_, sacrificed after day 1, focal degenerative and vascular alterations in the diaphragm muscle fibre could be seen. The microscopic findings were varied from a single, large perinuclear or polar vacuoles to diffuse, micronodular sarcoplasm vacuolisation. In the majority of these affected myocytes nucleus was round and centrally located. In addition, small, hyperchromatic nucleus was present, too. In these areas, focal loss of cross-striations of myocytes was present, too. The early, focal, muscle changes were accompanied by the intensive dilatation of the blood vessels, focal haemorrhages and perivascular accumulation of neutrophils and macrophages. Described pathohistological changes were the strongest in the diaphragm of rats treated with K048 or K075 (Fig. 3b and c). The DDS value of the K048-treated was 2.63 ± 0.62, while in the K075-treated animals a mean DDS was no higher than 2.40 ± 0.62 (P < 0.001 vs. control and asoxime-treated groups). A mean DDS was less in the other oxime-treated groups, but significantly different from the control group of animals (P < 0.001). The frequency and severity of the myocyte degeneration, intensity of haemorrhages and inflammatory cell infiltrate were significantly different on the seventh day of study in comparison to the same groups of animals, which were sacrificed 24 hrs after treatment. The time-dependent differences between established DDS values were the highest in the asoxime, obidoxime, and K027 and K075-treated groups, respectively (P < 0.001) (Table 3b).

In animals treated with a single injection of a median lethal dose of different oximes, diffuse micronodular degeneration with karyorrhexis or karyolysis was observed after 24 hrs. Affected muscle fibres were surrounded by massive, focal haemorrhages and increased number of inflammatory cell infiltrates. The interstitial haemorrhages appeared uniformly in each of the sections examined. The strongest diaphragm damages were shown in the groups of rats treated with obidoxime, K048 and K075, respectively. These DDS values were significantly different from those obtained in asoxime-treated animals (P < 0.001). The later phase of myocyte injury was characterized by multifocal necrosis and diffuses haemorrhages. The affected areas were invaded by neutrophils and numerous macrophages. Within 7 days, in the affected areas cross-striations were completely lost. Thickening of blood vessels with vacuolisation of the endothelial cells was observed, too. At the end of this experimental period, the strongest muscle injury was in the groups of animals treated with obidoxime, K048 and K075, respectively (Fig. 3d–f). Their DDS values were significantly higher than those obtained in the asoxime-treated groups and in the control ones (P < 0.001). On the other hand, the established time-depend differences were the most significant in the groups of rats treated with asoxime, obidoxime and K027, respectively (P < 0.001) (Table 3c).

### Pathohistological and semiquantitative analysis of the musculus popliteus of the experimental animals

**Figure 4:**
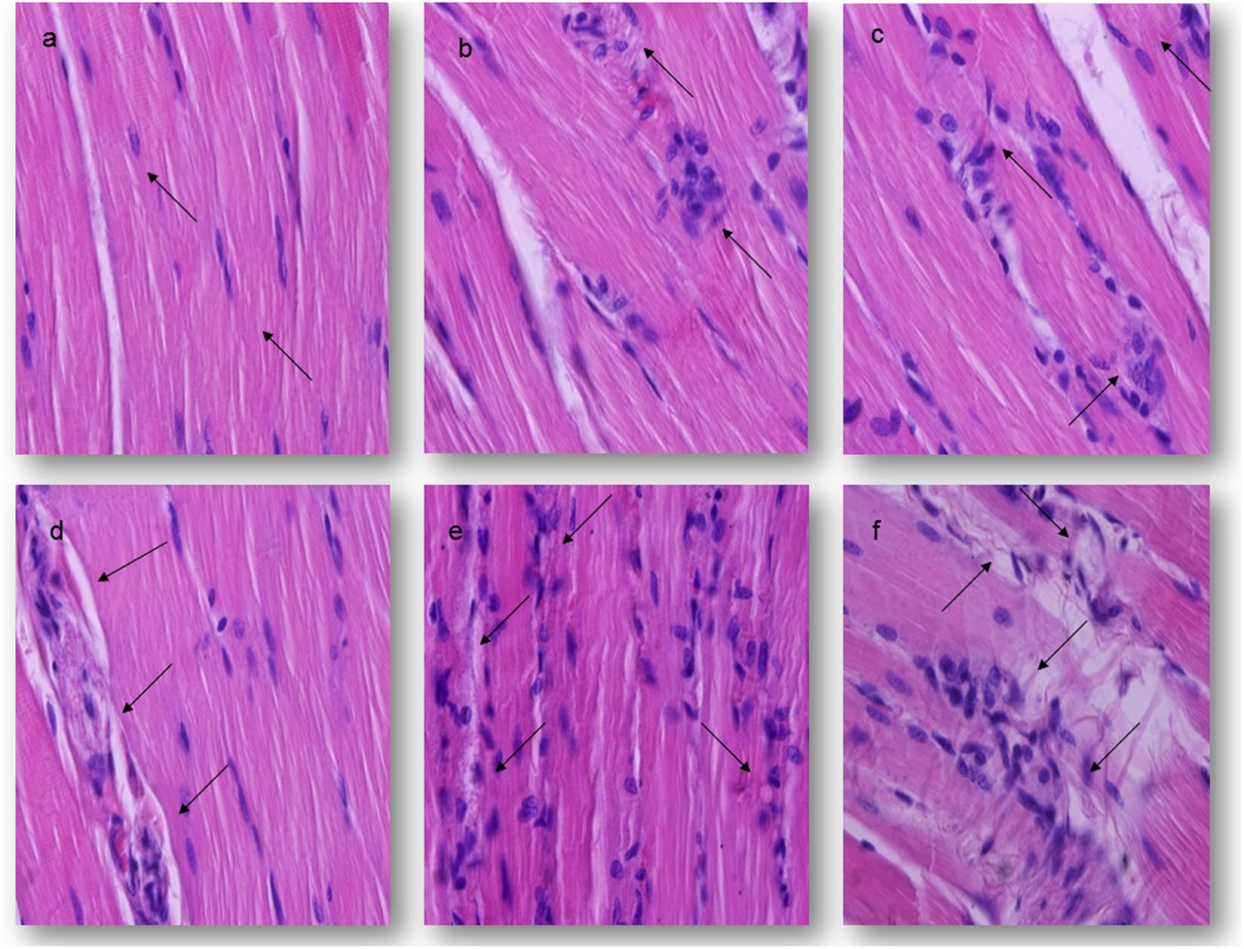
Light micrographs of the musculus popliteus lesions of rats 7 days after treatments. H&E stain. Magnification 400×. (**a**,**b**) −0.5 LD_50_ *im*; (**c**–**e**) −1.0 LD_50_ *im*; (**a**) The control group, normal histological structure of the musculus popliteus; (**b**) The K048-treated group, vacuolar degeneration of myofiber and focal perivascular accumulation of neutrophils and macrophages; (**c**) The K075-treated group, small necrotic foci surrounded with a large number of neutrophils and macrophages; (**d**) The asoxime-treated group, myofibrils lysis, cellular infiltration and interstitial edema; (**e**) The K048-treated group, hyalinization with massive and diffuse cellular infiltration; (**f**) The K075-treated group, fragmented necrotic muscular fiber surrounded by leucocytes, neutrophils and macrophages.

The histological structure of the musculus popliteus in the groups of animals treated with 0.1 LD_50_ of different oximes was similar to the control group (Fig. 4a). Some myofibrils cells with intracellular oedema and normal nuclei were seen. The structural integrity of the blood vessels wall was unchanged. The results presented in Table 4 clearly show that the MPDS values in rats treated by different oximes were very similar to the ones established in the control group, during the whole study. In addition, the difference between the MPDS values was not significantly different in these animals sacrificed on day 1 and 7 after treatment (Table 4a).

**Table 4:** The effects of different oximes (0.1 LD_50_, 0.5 LD_50_ or 1.0 LD_50_) on the degree of muscle injury - musculus popliteus damage score (MPDS) 24 hrs and 7 days after their administration.

Treatment	Days after treatment	MPDS (5 muscles/group × 6 slices/muscle) $\bar{{\boldsymbol{\times }}}$ ± S.D.		
		a) 0.1 LD_50_	b) 0.5 LD_50_	c) 1.0 LD_50_
control	1	0.17 ± 0.38	0.17 ± 0.38	0.17 ± 0.38
	7	0.17 ± 0.38	0.17 ± 0.38	0.17 ± 0.38
asoxime	1	0.17 ± 0.38	1.46 ± 0.63 **a**^**3**^	1.80 ± 0.76 **a**^**3**^
	7	0.20 ± 0.41	2.43 ± 0.52 **a**^**3**^**c**^**3**^	3.03 ± 0.61 **a**^**3**^**c**^**3**^
obidoxime	1	0.13 ± 0.35	1.30 ± 0.47 **a**^**3**^	2.67 ± 0.88 **a**^**3**^**b**^**3**^
	7	0.27 ± 0.45	2.20 ± 0.61 **a**^**3**^**c**^**3**^	2.67 ± 0.62 **a**^**3**^
K027	1	0.17 ± 0.38	1.10 ± 0.76 **a**^**3**^	1.50 ± 0.73 **a**^**3**^
	7	0.20 ± 0.41	2.10 ± 0.66 **a**^**3**^**c**^**3**^	2.76 ± 0.61 **a**^**3**^**c**^**3**^
K048	1	0.27 ± 0.45	2.03 ± 0.76 **a**^**3**^	3.23 ± 0.82 **a**^**3**^**b**^**3**^
	7	0.23 ± 0.43	3.10 ± 0.51 **a**^**3**^**c**^**3**^	3.90 ± 0.78 **a**^**3**^**c**^**1**^
K075	1	0.37 ± 0.61	2.96 ± 0.56 **a**^**3**^**b**^**3**^	3.57 ± 0.63 **a**^**3**^**b**^**3**^
	7	0.27 ± 0.45	4.20 ± 0.63 **a**^**3**^**b**^**3**^	4.40 ± 0.72 **a**^**3**^**b**^**3**^**c**^**2**^

Statistical evaluation was performed using Kruskall-Wallis test. **a**^**3**^ - P < 0.001 vs. control group; **b**^**3**^ - P < 0.001 vs. asoxime-treated group; **c**^**1**^,**c**^**2**^,**c**^**3**^ - P < 0.05, 0.01, 0.001 vs. 24 hrs.

The degenerative process was detected in all animals treated with 0.5 LD_50_ of different oximes. The most common findings were granular degeneration of the sarcoplasm, picnotic nucleus, vasodilatation associated with a various number of the haemorrhagic foci and perivascular accumulation of neutrophils and macrophages, and oedema and hyperaemia of the endomysium and the perimysium (Fig. 4b and c). Small necrotic foci could be seen, especially in the K075-treated animals, during the whole experiment. The highest MPDS value (4.20 ± 0.63) was established in the K075-treated rats sacrificed after 7 days. These values were significantly different from those obtained in the asoxime treated groups or in the control ones, sacrificed after day 24 hrs or 7 days. On the other hand, the intensity of the muscular lesions significantly increased with time over a period of 1 to 7 days in all oxime-treated groups (P < 0.001) (Table 4b).

A single administration of 1.0 LD_50_ of different oximes caused severe, diffuse and massive degenerative and vascular alterations in all tested groups. Affected muscle fibres appeared to be swollen and hypereosinophilic with loss of cross-striations. In the majority of this irregular, rounds to ovoid cell’s nucleus polymorphism were present, with large, round to rectangular shapes and prominent nucleolus. The altered contractile materials frequently were fragmented into large blocks or disks scattered along the “tube” of persisting external lamina of the muscle fibre. Within 24 hours, the affected areas were invaded by occasional neutrophils and numerous macrophages. Thickening of blood vessels with vacuolisation of the endothelial cells was observed, too. The massive, diffuse haemorrhage and interstitial oedema were accompanied by multifocal areas of the muscle fibre necrosis. The interstitial haemorrhage appeared uniformly in each of the sections examined. Observed muscle alterations were the most intensive in the K048 and K075-treated animals, during the whole experiment (Fig. 4d–f). In these groups, the MPDS values were significantly different from those established in the asoxime-treated or in the control rats (P < 0.001). During the experimental period of 7 days, the frequency and severity of the muscular alteration were the most significantly increased in the animals treated with asoxime and K027, respectively (P < 0.001) (Table 4c).

## Discussion

The determination of acute toxicity of each oxime is one of the major prerequisites for the development of newly synthesised oximes as therapeutic drugs. Evaluation of acute toxicity of any chemical compound plays an important role in the designing of drugs and their toxicological evaluation. The use of conventional toxicology studies with pathology is usually sufficient to predict important adverse drug effects and to support the safe use in further clinical trials^35^. The emphasis lies on the identification of pathological changes, the assessment of the relevance of these changes, and in particular the interpretation of animal tests in terms of human application (i.e. risk assessment)^35–37^. The promising approach for the determination of acute toxicity in experimental animals is by using a single dose of a chemical wherein lethality (i.e. LD_50_) can be determined within 24 hrsstudies^38,39^. In addition, the toxicity of a compound depends on their exposure routes, absorption, distribution, metabolism and reaction with the targeted tissues within the mammalian body.

Our study reports the acute toxicity of asoxime, obidoxime, K027, K048, and K075 using the intramuscular route of exposure in Wistar rats. The results showed that asoxime and K027 via *im* route had the least toxicity (626.38 mg/kg and 664.40 mg/kg, respectively) of all oximes. The rapid absorption of the drug via the muscles helps in the easy entry of the drug into the circulation which can thereby show the immediate effect. Also, oxime and K027 showed 3, 4 or 8 times less acute toxicity in comparison to K048, obidoxime and K075, respectively. Their values were also higher if compared with those previously obtained for obidoxime (211 mg.kg^−1^), pralidoxime (377.5 mg.kg^−1^) or K075 (49 mg.kg^−1^)^40^. Moreover, different exposure routes can have a different bioavailability of a drug which results in variable toxic effect within the same species. The large difference between the toxicity of each oxime via *ip* and *im* route underlays to the structural difference^38^. Then, the general toxicity of alkenes, alkynes and -cyclo compounds are greater than alkanes group^41^. Accordingly, the presence of more alkyl and quaternary nitrogen group contributes to the higher toxicity by obidoxime and K075, respectively^42^. This fact is in very good agreement with their acute toxicity data. It is known that oximes act also as acetylcholinesterase inhibitors. Some of them act as weak inhibitors (pralidoxime, K074, K075), some of them act as strong inhibitors (asoxime, obidoxime, K027, K048)^27,40^. By testing the therapeutic efficacy, all of K-oximes showed better antidotal activity than HI-6 at doses of 5% or 25% of their LD_50_^43^. Unfortunately, the higher acute toxicity of these oximes is a limiting factor for their usage. The first side effects without dead were noticed in rats 24 hrs after K027, HI-6 and obidoxime administration at a dose of 50% LD_50_^44^. For each investigative oxime, acute toxicities should be observed at dosages that exceeded pharmacologic efficacy by at least an order of magnitude^45^. Thanks to their LD_50_ and herewith obtained data, we can suggest that their acute toxicity is probably not caused by anticholinesterase activity, only.

Namely, different types of drugs cause muscle toxicity including statins, neuromuscular blocking agents, beta-adrenergic receptor agonists, anticonvulsants and morphine^45–48^. Furthermore, the incidence of skeletal muscle toxicity is increasingly more prevalent in the preclinical evaluation of new drugs^45,49–51^. There is not enough information available to assess the relationship between different oximes use and muscular damage. The pathogenesis of oxime-induced muscle toxicity is probably complex, for example, high doses of examinated oximes cause skeletal muscle injury. Moreover, exposure of muscle fibres to a dose of different oximes, higher than theirs 0.5 LD_50_, probably induced active muscle degeneration and inflammation occurs within the first few days after injury^52^.

In our experiment, the active muscle degeneration, congestion of the blood vessels, and neutrophil aggregation could be found in the heart, diaphragm and musculus popliteus of rats treated with 0.5 LD_50_ of obidoxime, K027, K048 and K075 observed on the day 1. A mild to moderate intensity of muscle injuries were found in all examinated tissue samples (a mean CDS, DDS, and MPDS were in the range within 1.67 to 2.16, 1.10 to 2.63, and 1.10 to 2.96, respectively). The most prominent increase of CDS, DDS and MPDS were noticed in all examinated muscle tissue treated by 1.0LD_50_ of obidoxime, K027, K048 and K075 (a mean CDS, DDS and MPDS were in the range 2.40 to 4.40, P < 0.001 vs. asoxime groups). At the highest concentrations, the initial event is necrosis of the muscle fibres, which is a consequence primarily by an unregulated influx of calcium through sarcolemma lesions^53^. This increase of cytoplasmic calcium causes proteases and hydrolases activation, which induces further muscle damages and intensive activation of enzymes that induce the production of mitogenic substances for muscle and immune cells^54^. Then, neutrophils infiltrating the muscle lesion and secrete a large number of proinflammatory molecules such as cytokines (TNF-α, IL-6), chemokine (CCL17, CCL2) and growth factors (FGF, HGF, IGF-I, VEGF, TGF-β1) in order to create a chemoattractive microenvironment for monocytes and macrophages infiltration^52,55–58^. During the first phase, within few days after drug-induced muscle injury, M1 as pro-inflammatory macrophages contribute to cell lysis, removal of cellular debris and stimulate myoblast proliferation^59–61^. Then, during the first week after muscle toxic injury, M2 or anti-inflammatory macrophages, attenuate the inflammatory response and participate in muscle regeneration process^62,63^ or to stimulate fibrosis^64,65^. It’s a multiple step process including activation and proliferation of the satellite cells (SC), repair and maturation of damaged muscle fibres and connective tissue formation^52^.

In our study, it was actually noticed after a period of 1 week by using semiquantitative histopathological analysis. Severe injuries of muscle fibres, followed by oedema, haemorrhages, neutrophil and macrophages infiltration, the proliferation of fibroblasts and blood vessels were found in all types of muscle fibres following treatment with 0.5 LD_50_ of obidoxime, K027, K048 and K075. The calculated values of CDS, DDS and MPDS were in the range of 2.10 to 4.20. Also, we found that registered scores in all muscle tissues were lowest on day 7 only in the asoxime-treated group (a mean CDS, DDS and MPDS were in the range of 2.07 to 2.43). Later on, calculated CDS, DDS and MPDS were increased following treatment with 1.0LD_50_ of asoxime but their values were significantly lower (P < 0.001) than in the other oxime-treated groups. The most severe muscle injuries were found on day 7 in rats treated with 1.0 LD_50_ of K048 and K075. The maximal calculated values of CDS, DDS and MPDS were in the range of 3.90 to 4.40.

Our own results, as well as others^45,52^, support the statement that this type of oxime-induced toxicity is dose-related, depending upon their rate of progression, they can cause acute toxic muscle injury which leads to drug-induced myopathies. Namely, muscle fibres are post-mitotic cells, which do not have the capacity to divide. Following an injury, damaged muscle fibres cannot be repaired without the presence of the satellite cells (SC)^66,67^. Following activation, adult muscle stem cells, SC, proliferate and generate a population of myoblasts that can either differentiate to repair damaged muscle fibres or self-renew to maintain the SC zone for possible future demands of muscle regeneration^68,69^. Also, satellite cells attract monocytes and use macrophages as a support to escape necrosis and enhance muscle growth^63^. This monocyte and macrophage populations were associated with the production of proinflammatory cytokines and removal of necrotic debris^60^.

In conclusion, our study confirms that oximes themselves could have a toxic effect on skeletal muscle fibres. The early muscle alterations observed in our study seem to contribute to the pathogenesis of the oxime-induced toxic muscle injury, which probably manifests as necrosis and/or inflammation. However, the exact cause-effect relationship causing cellular injury remains to be established.

## Methods

### Used chemicals

**Figure 5:**
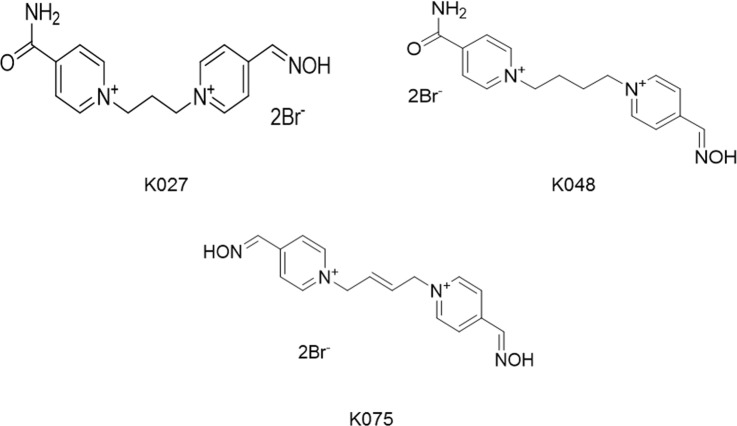
Chemical structure of tested K-oximes.

For this *in vivo* study, five AChE reactivators were used - asoxime, obidoxime, K027, K048, and K075 (Fig. 5).

They were prepared according to the synthetic approaches published earlier in the literature^70–72^. Their purity was tested before the experiment using standard analytical approaches^73,74^.

### Experimental animals

The experiment was performed on adult male Wistar rats 8 weeks old, weighing 180–220 g (Institute for medical research, Military Medical Academy, Belgrade, Serbia). The experimental animals were housed in plastic cages (Macrolon® cage type 4, Bioscape, Germany) with sawdust bedding (Versele-Laga, Belgium) certificated as having contaminant levels below toxic concentrations. The environmental conditions were controlled and monitored by a central computer-assisted system with a temperature of 22 ± 2 °C, relative humidity of 55 ± 15%, 15–20 air changes/h, and artificial lighting of approximately 220 lux (12 hrs light/dark cycle). The experimental animals had free access to food, commercial pellets for rats (Veterinarski Zavod Subotica, Serbia) and tap water from municipal mains, filtered through a 1.0 μm filter (Skala Green, Serbia).

All animal care and experimental procedures were approved by (i) the Ethics Committee for Animals Experiments of the Military Medical Academy, Belgrade, Serbia (approved document 282-12/2002); (ii) all experiments were performed in accordance with Guideline for Laboratory Animal Welfare, Ethics Committee for Animals Experiments of the Military Medical Academy, Belgrade, Serbia (decision No. 323-07-04943/2014-05/1) who was adopted in complete accordance with the current National Guidelines for Animal Welfare of the Republic of Serbia approved by the European Commission (published in the Official Gazette, Republic of Serbia, No. 41/2009).

### Acute toxicity

(i). Asoxime. Acute toxicity of asoxime was established by using four groups of experimental animals. Experimental groups consisted of 5 animals, each. Increasing doses of asoxime (500, 600, 700 and 800 mg/kg) were applied by *im* route, a separate groups of animals.
(ii). Obidoxime. Acute toxicity of obidoxime was recorded by using four group of rats, each of them consisted of 5 animals. Increasing doses of obidoxime (100, 150, 200 and 300 mg/kg) were administrated by *im* route in a separate group of animals.
(iii). K027. Acute toxicity of K027 was established by using four groups of experimental animals. Each experimental groups consisted of 5 animals. Increasing doses of asoxime (500, 600, 700 and 800 mg/kg) were applied by *im* route in a separate group of animals.
(iv). K048. Acute toxicity of K048 was tested by using four group of rats, each of them consisted of 5 animals. Increasing doses of K048 (100, 200, 300 and 400 mg/kg) were administrated by *im* route in a separate group of animals.
(v). K075. Acute toxicity of K075 was registered by using four group of rats, each of them consisted of 5 animals. Increasing doses of K075 (50, 75, 100 and 150 mg/kg) were administrated by *im* route, a separate group of animals.
(vi). Median lethal dose (LD_50_). After the 24-h survival registration, median lethal dose (LD_50_) for asoxime, obidoxime, K027, K048, and K075 was calculated according to the method of Litchfield & Wilcoxon^75^.

### Experimental design

Wistar rats were randomly divided into sixteen experimental groups each group containing ten individuals. The animals received the following treatments: (1) Control (0.9% saline 1 ml/kg *im*), (2) asoxime0.1LD_50_ (63 mg/kg *im*), (3) asoxime 0.5LD_50_ (313 mg/kg *im*), (4) asoxime 1.0LD_50_ (626 mg/kg *im*), (5) obidoxime 0.1LD_50_ (16 mg/kg *im*), (6) obidoxime 0.5LD_50_ (82 mg/kg *im*), (7) obidoxime 1.0LD_50_ (164 mg/kg *im*), (8) K027 0.1LD_50_ (66 mg/kg *im*), (9) K027 0.5LD_50_ (332 mg/kg *im*), (10) K027 1.0LD_50_ (664 mg/kg *im*), (11) K048 0.1LD_50_ (23 mg/kg *im*), (12) K048 0.5LD_50_ (115 mg/kg *im*), (13) K048 1.0LD_50_ (230 mg/kg *im*), (14) K075 0.1LD_50_ (8 mg/kg *im*), (15) K075 0.5LD_50_ (41 mg/kg *im*), and (16) K075 1.0LD_50_ (82 mg/kg *im*). Before administration, each oxime was diluted in freshly prepared solution of normal saline (sodium chloride 0.9 per cent in distilled water). Intramuscular administration of each oxime was carried out in lateral thigh muscle (i.e. *biceps femoris*) of the right leg at angle of 45° ^38^. The general condition of the animals was observed daily throughout the whole experiment lasting 7 days. Postmortem examination, heart, diaphragm and musculus popliteus of the left leg excision were also done at that time.

The selection of dose levels for detailed histopathological analysis was based on the results of acute toxicity testing, on range-finding studies, and on pharmacokinetic data^35^. For evaluation dose-depend and time-depend acute muscle toxicity, four dose groups of animals were included: a control group; a low-dose group (a dose that produces no compound related toxicity); a mid-dose group (a dose that elicits some minimal signs of toxicity); and a high-dose group (a dose that results in prominent toxic effects). Namely, the current legislation requires pathology expertise for test acute toxicity which meets all the proper requirements for good safety assessment of chemicals under investigation and does this according to the OECD Guidelines on a GLP-compliant way^35–37^.

### Histopathological procedure

In order to evaluate the effects of different oximes, five animals from each experimental group were sacrificed 1 and 7 days after receiving treatment under light ether anaesthesia.

At necropsy, the dissected heart, diaphragm and musculus popliteus were carefully spread over a metal tray coated with wax and fixed with 10% neutral buffered formalin solution. Five to seven days after fixation all tissues were divided into 6 portions in order to be prepared for making sections. After the process of fixation, all tissue samples were dehydrated in graded alcohol (100%, 96% and 70%), xylol and embedded in paraffin blocks. Finally, 2-μm thick paraffin sections were stained by haematoxylin and eosin (H&E) method and whole visual fields magnified by 200x were analysed.

### Semiquantitative analysis

The type, degree and severity of heart, diaphragm and musculus popliteus lesions along with the degree of inflammatory cellular infiltration were assessed in all tissue sections from each animal, and they were counted in separate visual fields at 400x magnification. The severity of heart, diaphragm and musculus popliteus lesions consisting of oedema, cellular infiltration, haemorrhages, vacuolar degeneration, necrosis, and the distribution of lesions (e.g., focal, multifocal, locally extensive, or diffuse) were assessed and graded by two independent pathologists. From each slice, whole visual fields were analyzed by using a light microscope according to the 5-point semiquantitative scale (Table 5) according to the degree and extent of the changes described above^76–79^. A severity grade was expressed as cardiac damage score (CDS), diaphragm damage score (DDS) and musculus popliteus damage score (MPDS), respectively. The exact way of their calculation is shown in Tables 2–4.

**Table 5:** Grading system for tissue lesions - cardiac damage score (CDS), diaphragm damage score (DDS) and musculus popliteus damage score (MPDS).

Grade	Definition
0	Normal histological structure.
1	Discrete intracellular oedema with the normal nucleus. A few neutrophils and macrophages.
2	Mild myofibril degeneration and normal nucleus architecture. Focal cellular infiltration.
3	Moderate myofibril degeneration. Discrete oedema and hyperaemia. Focal cellular infiltration.
4	Majority myocytes (more than 50%) with marked vacuolar degeneration and karyopicnosis. Focal haemorrhages. Focal cellular infiltration.
5	Myofibril lysis and hyalinization. Diffuse haemorrhages. Diffuse cellular infiltration.

### Statistical analysis

Statistical evaluation was performed using commercial statistical software (Stat for Windows, R.7, Stat Soft, Inc., USA, 2008). In tables, all results were showed as the mean ($\bar{{\boldsymbol{\times }}}$) ± the standard deviation (s.d.). Differences in tissue damage scores between groups were compared using the Kruskal-Wallis 1-way ANOVA test. All the analyses were estimated at minimal P < 0.05 level of statistical significance.

### Ethical approval

All animal care and experimental procedures were approved by (i) the Ethics Committee for Animals Experiments of the Military Medical Academy, Belgrade, Serbia (approved document 282-12/2002); (ii) all experiments were performed in accordance with Guideline for Laboratory Animal Welfare, Ethics Committee for Animals Experiments of the Military Medical Academy, Belgrade, Serbia (decision No. 323-07-04943/2014-05/1) who was adopted in complete accordance with the current National Guidelines for Animal Welfare of the Republic of Serbia approved by the European Commission (published in the Official Gazette, Republic of Serbia, No. 41/2009).
